# The effectiveness of one-to-one peer support in mental health services: a systematic review and meta-analysis

**DOI:** 10.1186/s12888-020-02923-3

**Published:** 2020-11-11

**Authors:** Sarah White, Rhiannon Foster, Jacqueline Marks, Rosaleen Morshead, Lucy Goldsmith, Sally Barlow, Jacqueline Sin, Steve Gillard

**Affiliations:** 1 Population Health Research Institute, St George’s, University of London, Cranmer Terrace, London, SW17 0RE UK; 2 School of Health Sciences, City, University of London, London, EC1V 0HB UK

**Keywords:** Peer support, Peer worker, Mental health services, Randomised clinical trial, Systematic review, Meta-analysis, Empowerment, recovery, Social network

## Abstract

**Background:**

Peer support is being introduced into mental health services internationally, often in response to workforce policy. Earlier systematic reviews incorporate different modalities of peer support (i.e. group and one-to-one), offer inconsistent evidence of effectiveness, and also indicate substantial heterogeneity and issues of quality in the evidence base at that time. An updated review, focussed on one-to-one peer support, is timely given current policy interest. This study aims to systematically review evidence for the effectiveness of one-to-one peer support interventions for adults using mental health services, and to explore heterogeneity in peer support interventions.

**Method:**

We searched MEDLINE, PsycINFO, Embase, CINAHL and Cochrane databases from inception until 13 June 2019. Included studies were assessed for risk of bias, and meta-analyses conducted where multiple trials provided usable data.

**Results:**

Twenty-three studies reporting nineteen trials were eligible, providing data from 3329 participants. While seven trials were of low to moderate risk of bias, incomplete reporting of data in many studies suggested bias in the evidence base. Peer support interventions included peer workers in paraclinical roles (e.g. case manager), providing structured behavioural interventions, or more flexible support for recovery.

Meta-analyses were conducted for eleven outcomes, with evidence that one-to-one peer support may have a modest positive impact on self-reported recovery and empowerment. There was no impact on clinical symptoms or service use. Analyses of heterogeneity suggest that peer support might improve social network support.

**Conclusions:**

One-to-one peer support in mental health services might impact positively on psychosocial outcomes, but is unlikely to improve clinical outcomes. In order to better inform the introduction of peer support into mental health services, improvement of the evidence base requires complete reporting of outcome data, selection of outcomes that relate to intervention mechanisms, exploration of heterogeneity in the implementation of peer support and focused reviews of specific types of one-to-one peer support.

**Trial registration:**

Prospero identifier: CRD42015025621.

**Supplementary Information:**

**Supplementary information** accompanies this paper at 10.1186/s12888-020-02923-3.

## Background

### Rationale

Mental health and workforce policies in a number of countries advocate the introduction of large numbers of peer workers into mental health services [1, 2]. In this context, peer workers are people with personal experience of using mental health services and/ or of mental distress, employed to make use of that experience in providing support to others currently using mental health services. Peer support more generally refers to a mutual exchange of emotional and practical support between people who identify as peers on the basis of shared or similar experiences of mental distress, with the recent origins of organised forms of peer support often ascribed to the mutual aid movement [3, 4]. The emergence of trained peer workers, providing peer support to people using mental health services, is a comparatively newer phenomenon, stimulated perhaps in part by assumptions about economic prudence [5], and in part by suggestions that peer support aids individual recovery [6]. Peer workers have been employed in a range of roles, providing one-to-one support to individuals using mental health services, as we explore below, supporting and facilitating mutual support groups, or running services provided as an alternative to mainstream provision.

The peer support literature has been reviewed before, with Pitt and colleagues [7] finding a small reduction in emergency service use where peer workers were compared with other mental health professionals working in similar roles (primarily case management), while Lloyd Evans and team [8] found a modest positive effect of peer-provided interventions on self-reported recovery and hope. However, both reviews combined studies of individual and group-based peer support – noting substantial heterogeneity in both intervention and trial population – and in both reviews authors cautioned that the majority of trials were of low to moderate quality and that reporting bias in particular might explain these results. More focused reviews have considered peer support for people experiencing depression [9], and for those experiencing psychosis [10]. The former considered only group interventions, while the latter combined group, one-to-one and service-level modalities of peer support, and found no evidence of effectiveness of one-to-one peer support. However, a recent, informal review has indicated that a number of new trials of one-to-one peer support in mental health services have been reported [11], offering a timely opportunity for a systematic review focusing on one-to-one peer support in order to provide an evidence base for the ongoing introduction of peer workers into mental health services internationally.

### Exploring heterogeneity of peer support interventions

We note that Pitt and colleagues [7] identified small differences in effect when considering ‘consumer provider [peer] vs professional staff’ in comparison to ‘consumer provider as an adjunct vs usual care alone’, warranting exploration of this aspect of intervention heterogeneity in the context of one-to-one peer support in this review. Both Pitt [7] and Lloyd Evans [8] also note that peer support is often under specified in trial papers, and that it is not always clear how peer support is different from mental health support provided by other types of mental health worker. A wider literature has identified a values-base that specifies how peer support is distinctive from other mental health support, suggesting that peer support is characterised by: a relationship grounded in a sense of connection based on shared experiences [12]; the use of experiential, rather than formal (taught) knowledge in the peer worker role [13]; the reciprocal nature of the relationship, with both parties learning from each other in contrast to the uni-directional clinician-patient relationship [14]. However, it is also noted how the formal, health services environment is not always conducive to the delivery of peer support [15, 16].

Studies have identified a number of organisational factors that facilitate the implementation of distinctive peer support into practice, including: a clear, shared understanding of the values informing peer support in the peer worker role [17]; the importance of dedicated peer support training programmes for peer workers [18]; the need for support and supervision for peer workers [19]. Some actors in the peer support community have called for standards in the delivery of peer support in mental health services to ensure that a distinctive, values-based peer support is delivered [20]. Currently there is a lack of evidence of any association between outcomes and organisational variables supporting the delivery of peer support. There is therefore a case for exploring whether it is possible to operationalise, as a subgroup analysis, the quality of organisational support for one-to-one peer support interventions as an additional approach to exploring the heterogeneity of peer support in mental health services.

This study aims to:

- systematically review all the available peer-reviewed evidence for one-to-one peer support interventions for adults using mental health services
- evaluate the effects of one-to-one peer support in mental health services on a range of pre-specified outcomes
- investigate, using subgroup analyses, how heterogeneity in intervention (i.e. type of peer support, quality of organisational support for peer support) is related to outcome.

## Methods

This systematic review and meta-analysis adheres to PRISMA guidelines and is funded by the UK National Institute for Health Research as part of larger programme of research investigating peer support in mental health services. The review protocol is registered with the International Prospective Register Of Systematic Reviews, identifier: CRD42015025621.

### Definitions

For the purpose of this review we consider one-to-one peer support in mental health services to be support delivered by an individual with personal experiences of using mental health services and/or of mental distress. We refer to the person delivering peer support here as a *peer worker*, noting that other terms, including *peer support worker*, *peer specialist* and *consumer employee*, have been used elsewhere. Peer workers are employed – whether paid or unpaid – and trained to make use of their experiential knowledge in providing support to someone who shares similar experiences, as part of or alongside the care and treatment they are receiving from mental health services.

#### Eligibility criteria

Studies were included where peer support was:

- provided one-to-one;
- intentionally provided by a peer worker;
- for adults using mental health services.

Studies were excluded if peer support was:

- not the primary means of delivering the intervention;
- not one-to-one or intentionally provided by a peer worker;
- where mental health was not the primary focus of the intervention.

Other exclusions were applied if the study was not in the English language, non-retrievable, or did not contain empirical data.

### Study design

All types of randomised controlled trial (RCT) were included. Other study types were excluded.

### Intervention and comparison conditions

We noted above that one-to-one peer support in mental health services has been characterised as either: an adjunctive intervention, delivered by peer workers in addition to care as usual or as an additional component to a treatment or therapy; or as peer workers delivering similar interventions to those delivered by other mental health workers (e.g. where peer workers are employed in a substitute capacity) [7]. We include both ‘adjunctive’ and ‘substitute’ peer support interventions in this review, and consider all comparator conditions in our primary analysis. Where trials had two or more intervention arms (e.g. with and without peer support) and a control arm, in all cases the comparison chosen was peer support (as intervention condition) and the other enhanced or active condition (as control condition), rather than care as usual or an attention control arm.

### Outcomes

As noted above, a variety of outcomes have been assessed in peer support trials. Given that a number of additional trials have emerged since the publication of existing systematic reviews, it is of interest to consider whether the range of outcomes of interest remains broad or has begun to coalesce. We extract data using the set of outcomes explored in the review conducted by Lloyd-Evans and colleagues [8]. In addition, we consider use of emergency services in order to explore further findings in the Pitt review [7] and, following other published research into the mechanisms of peer support we include a small number of more socially-focused outcomes that may be impacted by peer support [21]. The full set of outcomes of interest for this review is as follows:

- Hospitalisation
- Emergency service use
- Employment
- Overall psychiatric symptoms
- Symptoms of psychosis
- Depression and anxiety
- Quality of Life
- Recovery (self-rated)
- Hope
- Empowerment
- Satisfaction with services
- Social functioning
- Social network support
- Working alliance (clinician rated/ patient rated)
- Self-stigma
- Experienced stigma
- Engagement with services
- Wellbeing

#### Search methods for identification of studies

The following online bibliographic databases were identified in 2015 based on then existing reviews [6, 7] – Cochrane Central Register of Controlled Trials, MEDLINE, EMBASE, PsychINFO and CINAHL Plus – and searched initially from inception until the end of April 2015.

Existing reviews were used to provide a basis for search terms, with authors using their knowledge of the area, including service user researchers JM and RF, to add to search terms. The diagnostic manual DSM 5 [22] was consulted to provide a systematic structure to ensure mental health terms were inclusive. The search strategy was tested and refined as necessary. All databases were searched using a similar set of terms, strategies and Boolean operators, amended solely for the purposes of the research database management interface and not for content. An example of the search strategy, for MEDLINE, EMBASE and PsychINFO using the OVID interface, is given in Supplementary materials 1.

Searches were updated on 13 June 2019, with no changes to search terms or search strategy. All papers returned by the search were imported into an Endnote library and any duplicates removed first using the software and then by manual review.

### Study selection

Titles and abstracts of all studies returned in the search were independently screened for inclusion in the review by two researchers (two of JM, RF and RM). Disagreements were resolved by discussion using the full text of the paper, with remaining differences resolved by discussion with SG. Reference list and forward citation searching of included studies were used to identify additional papers for inclusion in the review.

#### Data extraction

Data were extracted for study characteristics from each included study by one of JM, RF or RM using a structured data extraction data sheet (see Table 1 below), with a second researcher (SG) checking for accuracy of extraction for 25% of studies.

**Table 1:** Detailed characteristics of studies

Study	Country	Method	Population	Sample size: intervention /control	Intervention	Control	Outcomes	Assessments	Longest Follow Up	Type of PS	Support for PS
Solomon, 1995 [23]	USA	Randomised controlled trial	Adults currently on community mental health centre caseload who meet all three criteria for intensive case management and were identified to be at risk for hospitalisation with a diagnosis of major mental illness and a significant treatment history	48/48	Consumer case management	Case management as usual from community mental health services	1) Overall psychiatric symptoms^b^ 2) Social network support ^b^ 3) Quality of life ^b^ 4) Hospitalisation^b^ 5) Working Alliance	1) Brief Psychiatric Rating Scale (BPRS) [24]^b^ 2) Pattison’s Social Network [25]^b^ 3) Lehmans’s Quality of Life Interview [26]^b^ 4) Days in hospital^b^ 5) Working Alliance Inventory - staff and client [27]^b^	24 months	S	H
Klein, 1998 [28]	USA	Randomised controlled trial - pilot	Adult patients receiving intensive care management with dual diagnosis who had been in community care at the mental health centre for 1 year	10/51	Peer-supported community enablement plus CAU	CAU - Intensive Case Management	1) Hospitalisation 2) Social functioning 3) Quality of Life 4) Social network support 5) Wellbeing	1) Days in hospital 2) Global Assessment of Functioning (GAF) Scale [29] 3) Lehman’s Quality of Life (QOL) [26] 4) Lehman’s Quality of Life – Friends subscale [26] 5) Lehman’s Quality of Life – Health subscale [26]	6 months	A	H
Clarke, 2000 [30]	USA	Randomised controlled trial – three arms	Adult patients with a severe mental disorder, a schizophrenic, major affective, or paranoid disorder, or another severe mental disorder, and a documented history of persistent psychotic symptoms other than those caused by substance abuse.	57/57/49	Consumer-staffed assertive community treatment	1) Non-consumer assertive community treatment^a^ 2) CAU – usual community mental health services	1) Hospitalisation^b^	1) Hospitalised^b^ Community tenure (days)	6 months	S	L
Hunkeler, 2000 [31]	USA	Randomized controlled trial – three arms	Adults primary care patients with a diagnosis of major depressive disorder or dysthymia and given a prescription of for a SSRI antidepressant (fluoxetine hydrochloride or paroxetine)	123/117/62	Peer support via telephone contact or face-to-face plus nurse telehealth care plus nurse telehelthcare plus CAU	1) Nurse teleheath care plus CAU 2) CAU – usual physician care	1) Depression and anxiety 2) Social functioning 3) Satisfaction with services	1) Hamilton Depression Rating Scale- self report version [32] - Beck depression Inventory [33] 2) SF − 12 Mental and Physical Composite Scales [34] 3) Patient satisfaction with treatment scale – no information provided	6 months	A	L
Craig, 2004 [35]	England	Randomised controlled trial - pilot	Adult service users currently registered with assertive outreach team and have SMI, with a record of poor engagement, multiple hospitalisations and a high prevalence of problematical behaviours and substance abuse.	24/21	Consumer Health Care Assistant plus CAU	CAU - case management from Assertive Outreach Team	1) Social functioning ^b^ 2) Social network support ^b^ 3) Hospitalisation^b^ 4) Satisfaction with services 5) Service engagement	1) Life Skills Profile [36]^b^ 2) Significant others scale (SOS) [37] 3) Days in hospital^b^ Hospitalised^b^ 4) Verona Service Satisfaction Scale (VSSS) [38]^b^ 5) Number of missed (DNA) appointments with services.	12 months	A	H
Sells, 2006 [39]	USA	Randomised controlled trial	Adult patients currently using local mental health authorities with a primary diagnosis of SMI (schizophrenia spectrum disorder, major mood disorder, or both) and treatment disengagement	58/59	Peer-based case management from peer mental health service provider	Case management as usual from assertive community treatment teams	1) Working alliance - client^b^ 2) Engagement with services	1) Barrett-Lennard Relationship Inventory (BLRI) modified version [40]^b^ 2) Level of Care Utilization System [41]	12 months	A	L
Rivera, 2007 [42]	USA	Randomised controlled trial – three arms	Adults recruited from inpatient units at a city hospital whom have a diagnosis of a psychotic or mood disorder on axis I, and have had two or more psychiatric hospitalizations in previous two years	70/66/67	Consumer-assisted intensive case management	1) Intensive case management^a^ 2) Standard case management (i.e. office-based without intensive components)	1) Overall psychiatric symptoms^b^ 2) Quality of Life^b^ 3) Social network support^b^ 4) Wellbeing^b^ 5) Hospitalisation^b^	1) Brief Symptom Inventory [43]^b^ 2) Lehman Quality of Life Inventory [26]^b^ 3) Modification of Pattison Network Inventory [25]^b^ 4) Lehman’s Quality of Life - health subscale [26]^b^ 5) Days in hospital (per month)^b^	12 months	A	L
Simon, 2011 [44]	USA	Randomised controlled trial	Participants, aged 19 or over, who were currently in treatment for bipolar disorder	64/54	Online peer recovery coaching plus online recovery planning	Online recovery planning	1) Engagement with services	1) Use of online program components - engagement with recovery plans, use of social networking features, use of self-monitoring tools.	3 weeks	A	H
Sledge, 2011 [45]	USA	Randomised controlled trial - pilot	Adult inpatients who have experienced three or more psychiatric hospitalizations (or two admissions plus more than three psychiatric ED visits) during the 18-month period prior to recruitment and have a documented diagnosis of schizophrenia, schizoaffective disorder, psychotic disorder not otherwise specified, bipolar disorder or major depressive disorder with or without psychotic features	48/45	Community-based peer recovery mentor plus CAU	CAU - community mental health care	1) Hospitalisation^b^ 2) Overall psychiatric symptoms 3) Social Functioning 4) Hope 5) Satisfaction with services 6) Social network support 7) Wellbeing	1) No. of readmissions - Days in hospital^b^ Hospitalised^b^ Community tenure 2) Brief Psychiatric Rating Scale (BPRS) [24] 3) The Social Functioning Scale [46] 4) The Dispositional Hope Scale [47] 5) Mental Health Statistics Improvement Programme (MHSIP) [48] 6) Sense of Community Index [49] 7) 36 item Short Form Health Survey (SF-36) [34]	9 months	A	H
Proudfoot, 2012 [50]	Australia	Randomised controlled trial – three arms	Adults diagnosed with bipolar disorder by a health professional within the past 12 months and currently being treated	134/139/134	Online peer coaching plus online psycho-education programme	1) Online psycho-education programme 2) attention control	1) Depression and anxiety 2) Social functioning 3) Empowerment	1) Goldberg Anxiety and Depression Scale (GADS) [51] 2) Work and Social Adjustment Scale [52] 3) Multi-dimensional Health Locus of Control [53]	6 months	A	L
Chinman, 2013 [54]	USA	Cluster randomised controlled trial	Current adult VA intensive case management patients who have had 30 psychiatric inpatient days or 3 psychiatric admissions in the past year with an Axis 1 psychiatric disorder.	252/216	Floating, additional peer-supported case management plus CAU	CAU - case management from community-based Intensive Case Management services	1) Quality of Life^b^ 2) Recovery^b^ 3) Empowerment^b^ 4) Overall psychiatric symptoms^b^	1) Lehman’s Quality of Life Interview [26]^b^ 2) The Mental Health Recovery Measure (MHRM) [55]^b^ Illness Management and Recovery Scale (IMR Scale) [56] 3) Patient Activation Measure [57]^b^ 4)BASIS-R [58] ^b^	QoL - 6 months Other - 12 months	A	H
Simpson, 2014 [59]	England	Randomised controlled trial - pilot	Inpatients, aged 18–65, approaching discharge/extended leave from acute mental health inpatient unit	23/23	Peer support plus CAU	CAU - community mental health services	1) Hope 2) Quality of Life 3) Hospitalisation^b^	1) Beck Hopelessness Scale (BHS - 20 item) [60] 2) EuroQol (EQ-5D) [61] 3) Hospitalised^b^	3 months	A	H
Wrobleski, 2015 [62]	Canada	Randomized controlled trial - pilot	Adult patients receiving care from a community mental health service with a persistent mental illness, that is significantly affecting daily functioning or a person with both a mental health diagnosis and substance use issue	12/9	Peer-supported self-management plus occupational therapy	Self-management support from a (non-peer) mental health worker plus occupational therapy	1) Quality of Life	1) Lehman’s Quality of Life Interview [26]	6 months	S	H
Rogers, 2016 [63]	USA	Randomised controlled trial	Clients, over the age of 18, who were court ordered for treatment because of a psychiatric crisis civilly committed for a mental health crisis, adjudicated by the state court to meet the definition of “a person with a serious mental illness,”	63/50	Individual peer-supported social inclusion and recovery support plus CAU	CAU - Peer-provided services (excluding individual peer support; e.g. social activities, educational courses, group peer support)	1) Social network support 2) Overall psychiatric symptoms 3) Recovery 4) Quality of Life	1) Interpersonal Support Evaluation List [64] 2) BASIS-R [58] 3) Recovery Assessment Scale [65] 4) Brief Quality of Life (BQOL; Lehman, 1988) [26]	6 months	A	H
Salzer, 2016 [66]	USA	Randomized controlled trial	Patients, aged 18 and above, using community outpatient mental health programmes with a diagnosis on the schizophrenia spectrum, bipolar disorder, or major depression	50/50	Peer-delivered support for independent living plus CAU	CAU - usual outpatient mental health care	1) Quality of life^b^ 2) Recovery ^b^ 3) Empowerment^b^ 4) Working Alliance	1) Lehman’s Quality of Life Interview [26]^b^ 2) Recovery Assessment Scale [65]^b^ 3) The Empowerment Scale [67]^b^ 4) Working Alliance Inventory [27]	12 months	A	H
Seeley, 2016 [68]	USA	Randomized controlled trial - pilot	Patients, aged 55 and above, referred to an intergovernmental agency and meeting criteria for mild to moderate depression and/or anxiety	31/31	Peer-supported cognitive behavioural intervention for mild-moderate depression and/ or anxiety	Waitlist control	1) Depression ^b^ 2) Anxiety 3) Working Alliance	1) PHQ-9 [69]^b^ 2) GAD-7 [70] 3) Working Alliance Inventory [27]	2.5 months	A	L
Mahlke, 2017 [71]	Germany	Randomised controlled trial	Patients, aged 18–80, using in- and out-patient services with primary diagnosis of schizophrenia and related disorders, affective disorders, or personality disorder and a duration of illness of more than 2 years.	114/112	Community-based peer support for individual recovery plus CAU	CAU - in-patient and out-patient mental health as usual	1) Overall psychiatric symptoms^b^ 2) Quality of Life^b^ 3) Social functioning^b^ 4) Empowerment^b^ 5) Hospitalisation^b^	1) Clinical Global Impression – Severity scale [72] 2) Modular System for Quality of Life and EuroQol Questionnaire EQ. 5D [61]^b^ 3) Global Assessment of Functioning (GAF) Scale [29]^b^ 4) General Self-Efficacy Scale [73]^b^ 5) Days in hospital^b^ Hospitalised^b^	12 months	A	H
Yamaguchi, 2017 [74]	Japan	Randomized controlled trial	Patients, age 20 years or older, using outpatient psychiatric clinic or psychiatric hospital in Tokyo, who received services from case managers in either a psychiatric day care or visiting nurse program.	26/27	Peer supported shared decision-making plus CAU	CAU - medical consultation	1) Overall psychiatric symptoms^b^ 2) Social Functioning^b^ 3) Empowerment^b^ 4) Working Alliance^b^	1) The Brief Psychiatric Rating Scale (BPRS) [24]^b^ 2) Global Assessment of Functioning (GAF) Scale [29]^b^ 3) Patient Activation Measure [57]^b^ 4) Scale To Assess Therapeutic Relationships in Community Mental Health Care (STAR) – Clinician & Patient versions [75] ^b^	12 months	A	L
Johnson, 2018 [76]	England	Randomised controlled trial	Adult patients currently on the caseload of crisis resolution teams for at least a week because of a psychiatric crisis	221/220	Peer-supported self-management plus CAU	CAU – community mental health services plus self-management workbook	1) Overall psychiatric symptoms^b^ 2) Social network support ^b^ 3) Recovery ^b^ 4) Satisfaction with services^b^ 5) Hospitalisation	1) Brief Psychiatric Rating Scale [24]^b^ 2) Lubben Social Network Scale [77]^b^ 3.a) Illness Management & Recovery Scale ^b^ (patient version) [56] 3.b) Questionnaire on the Process of Recovery (QPR) [78] 4) Client Satisfaction Questionnaire [79]^b^ 5) Community tenure (days)	18 months	A	H

Key: *PS* Peer Support, *A* Adjunctive, *S* Substitute, *L* Low level of organisational support for peer support, *H* High level of support for peer support; ^a^ Comparator included in meta analysis; ^b^ Outcome/ assessment included in meta analysis

For the purposes of exploring heterogeneity of intervention as subgroup analyses in the meta-analysis we also recorded where peer support was provided as an adjunctive intervention and where peer workers were working in a substitute role, as defined above, and in addition rated the quality of organisational support provided for peer support. To do this, studies were independently coded by two members of the team (JM and RM) where they reported any of the following indicators:

A. Dedicated peer support training;
B. Clear description of theory, processes or understanding of peer support;
C. Support structures for peer workers (e.g. supervision).

Discrepancies between researchers were discussed until agreement was reached. Studies were then categorised as having a ‘higher level’ of organisational support for peer support if they fulfilled at least two of the three indicators, or ‘lower level’ if they met one or less indicators.

### Extraction of data for meta-analysis

One researcher (RM) extracted data for outcomes onto a bespoke extraction sheet. Data were included if they were assessed using a standardised measure or, in the case of service use data, captured in clinical records. For continuous outcomes, sample sizes, mean and standard deviations by arm were extracted, and for dichotomous outcomes, the number of events and sample size per arm were extracted. All outcome data extraction was checked by statistician SW for accuracy and completeness. If data for a particular outcome were only reported by a single paper that outcome was not included in the meta-analysis. We wrote to authors of included studies for additional information and trial data where it was not included in the published article.

Where outcome data were reported for more than one follow-up point, the longest timepoint was used. Where more than one measure was used to report the same outcome in a study, we included the measure more commonly reported by other studies in the analysis.

#### Assessment of risk of bias

Each included study was assessed for risk of bias by two researchers (RM, JM), with any differences in assessment resolved by a third researcher (SW), in accordance with *Cochrane Collaboration Risk of Bias Tool* [80]:

- adequate sequence generation (selection bias)
- allocation concealment (selection bias)
- blinding of outcome assessment (detection bias)
- incomplete outcome data (attrition bias)
- selective outcome reporting (reporting bias)

It is important to note that although blinding of participants to allocation is usually assessed, in this particular instance the nature of a peer intervention means that all trial participants are unblinded. As such this particular source of bias is not assessed in this review, in line with existing reviews of peer support.

#### Statistical analysis

Effect sizes for continuous data were calculated as standardised mean difference (SMD), Hedges’ g, with studies weighted using the inverse variance method [81]. Risk ratios were calculated for dichotomous outcomes, and studies combined again using the inverse variance method. All pooled effect sizes are reported with 95% confidence intervals calculated using random-effects models. We used intention to treat data in all analyses.

Statistical heterogeneity was assessed through the I^2^ statistic which describes the percentage of the variability in effect estimates that is due to heterogeneity rather than chance and the *p*-value of the χ^2^ test (Q) for heterogeneity. A p-value < 0.10 and an I^2^ > 50% suggests substantial heterogeneity. Where substantial heterogeneity of effect sizes across trials is observed, subgroup analyses were conducted, comparing studies where:

1. peer support was provided as an adjunctive intervention, against those studies where peer workers were working in a substitute role;
2. a higher level of organisational support for peer support were reported, against those studies where a lower level was reported.

Differences between subgroups of studies were tested using the Qint test for heterogeneity, testing if effect sizes differ across subgroups. Review Manager (RevMan 5.2 for Windows) software [82] was used to conduct the meta-analyses.

## Results

**Fig. 1:**
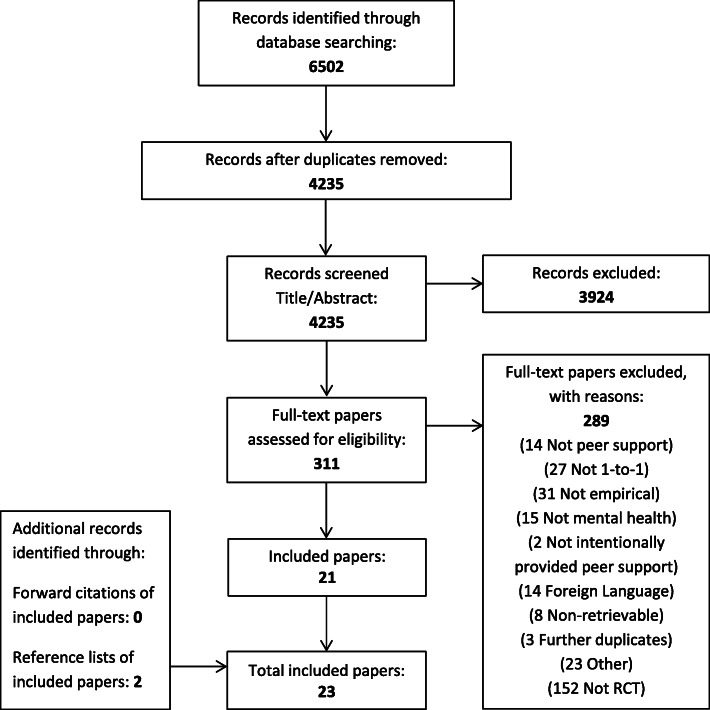
Flowchart of inclusion of studies

A total of 6502 records were identified in the updated search. Of these, 311 studies were potentially eligible and, after further review (as described above) 23 eligible papers were identified, reporting on 19 trials. One trial was reported across four papers [23, 83–85] and another trial reported across two papers [45, 86]. See Fig. 1 below.

### Study characteristics

Twelve studies were conducted in the USA [23, 28, 30, 31, 39, 42, 44, 45, 54, 63, 66, 68], three were conducted in the UK [35, 59, 76], one in Canada [62], one in Australia [50], one in Germany [71], and one in Japan [74]. Eighteen trials were individually randomised and one was a cluster randomised trial [54]. Six studies described themselves as pilot trials [28, 35, 45, 59, 62, 68], four studies were three-arm trials [30, 31, 42, 50], and one study used a waitlist control [68].

Fifteen studies fell into the ‘adjunctive’ peer support group, with eleven of those comparing peer support as an adjunctive intervention to care as usual [28, 31, 35, 42, 45, 54, 59, 66, 71, 74, 76]. Two of the eleven [31, 42] were three arm trials comparing care as usual, an adjunctive intervention, and the intervention plus an additional peer support component. Another three-arm trial [50] compared an attention control, intervention, and the intervention plus an additional peer support component. Two papers reported two arm trials of an intervention, with and without adjunctive peer support [44, 63], and one study compared the peer support intervention with a waitlist condition (i.e. no-comparator intervention) [68]. Four studies compared peer workers working in a substitute capacity compared with other mental health workers performing a similar role [23, 30, 39, 62]. One of these studies was also a three-arm trial [30], with care as usual as the third arm. Further details about study characteristics can be seen in Table 1 below, with indication given of which comparators were used in the subsequent meta-analysis.

### Population

Participants in all studies were adults, although in one study participants were aged 55 or older [68]. In the majority of studies – twelve – participants were using community mental health services [23, 28, 30, 35, 39, 44, 50, 54, 62, 63, 66, 76]. In three studies participants were recruited as inpatients [42, 45, 59], and in two studies participants were recruited as either inpatients or outpatients [71, 74]. In two studies participants were recruited directly from depression clinics [31, 68]. Most studies indicated diagnostic inclusion criteria, with seven studies specifying that participants would have diagnoses of either psychotic, or major depressive or mood disorders [23, 30, 39, 42, 45, 54, 66]. Two studies specified a diagnosis of bipolar disorder [44, 50], one of major depressive disorder [31], one of mild to moderate depression and anxiety [68], one of either psychotic or personality disorders [71], and one of dual mental health, and drug or alcohol disorder [28]. Two studies defined eligibility by duration of mental illness with one specifying at least two years [71], and the other indicating that mental illness should be persistent [62]. A number of studies defined the population by service use history. In three studies eligibility criteria were defined by a minimum number of previous, recent psychiatric hospital admissions [42, 45, 54]. One study recruited participants as they approached hospital discharge [59], one study recruited participants who had been referred to specialist crisis and home treatment teams [76], and another study recruited participants who were under a court order mandating community mental health treatment [63].

#### Sample sizes

Samples sizes in the studies ranged from 21 [62] to 468 [54], with a total of 3329 participants in the 19 trials.

#### Interventions

While descriptions of peer support interventions remains thin in some studies published since the last review [8], a number of more recent studies do provide detailed descriptions of peer worker roles and what constitutes peer support. Peer workers were reported as delivering a range of different interventions. Five studies reported peer workers working in case management roles [23, 30, 39, 42, 54]. Typically, peer workers were expected to fulfil a similar, brokerage-type case management function to other mental health workers, and in addition, to role model their own strengths and experiences of recovery [39], or to provide social support by arranging social activities [42]. Three studies reported peer workers working in mentoring or coaching roles [44, 45, 50]. Mentoring and coaching roles varied considerably from offering a very loosely described partnership relationship that aimed to be different to a clinician-patient relationship [45], to structured online coaching to support participants in producing a detailed, behaviourally-informed recovery plan [44]. Three studies described peer workers providing support for self-management [31, 62, 76]; for example, in one study peer workers provided one-to-one assistance with rehabilitation goals set by occupational therapists [62], while in another peer workers supported participants to complete a structured recovery workbook [76]. Another three studies describing peer workers offering support for recovery [59, 63, 71]. What support for recovery entailed was generally poorly defined, with the exception of Mahlke and colleagues [71], describing in some detail how peer workers were trained and supported to reflect on and make use of their own experiences as a resource in supporting others with their recovery, but also reported that the intervention was not further manualised, and that peer workers had flexibility in the role, with an emphasis on enhancing the sense of control over their lives that people experienced. Two studies reported peer workers providing support for living independently in the community [28, 66]. Peer support in both studies had a strong social focus and in the case of the latter [66], support was highly individualised and self-directed, involving the peer worker helping the individual to access social support that they identified themselves. Other studies described peer workers providing support for shared decision making in clinical consultations, again with a strong focus on a structured self-management approach [74], delivering a cognitive behavioural intervention using a structured workbook [68], and working in a healthcare assistant role [35].

Most peer support was provided face-to-face but in one study peer support was provided either face to face or by telephone [31], and in two studies peer support was provided online [44, 50]. We note that in three studies peer workers were employed by peer-led organisations or agencies [23, 63, 66]. As noted above, four studies evaluated peer workers as a substitute for other mental health workers working in a similar role, three of those in a case management capacity [23, 30, 39], and in the fourth, providing support for self-management [62]. In all other studies peer support was adjunctive to care as usual or evaluated as an enhancement to another intervention.

#### Level of support for peer support interventions

The majority of studies – fourteen and thirteen respectively – described the support and/ or supervision provided to peer workers to deliver the peer support intervention [23, 28, 35, 42, 44, 45, 54, 59, 62, 63, 66, 68, 71, 76], and the peer support-specific training provided to peer workers [31, 35, 39, 44, 45, 54, 59, 62, 63, 66, 71, 76, 85]. In contrast, only five studies explicitly described the theory, processes or understandings of peer support that underpinned the intervention evaluated [23, 28, 35, 54, 71].

There was variation in the degree of reporting of support given to peer workers. Reporting of training provided varied from noting that peer workers had received accredited peer specialist training prior to delivering the intervention [44], to a more detailed description of an extended, structured training program describing module content and mode of delivery [71]. Description of the support and supervision provided for peer workers also varied, from studies that simply reported that peer workers were provided with support and supervision for the duration of the study [59], to one which described in some detail the areas covered during weekly, 90 min group supervision sessions for peer workers [45]. One study said that supervision was provided by a peer support coordinator, with preference being given to employing someone with lived experience of mental illness in that role [62], while another stated that the director of the consumer case manager team was a consumer [23]. However no studies clearly stated that supervision for peer workers was provided by someone who was themselves employed to use their personal experiences of mental distress or of having used mental health services in the role. Theory, processes and understanding of peer support also varied in description, with one study [71] describing a specific peer support change model that underpinned the intervention, while others gave a more general description of the processes that characterise peer support as distinctive from other forms of mental health support [35].

Three studies did not report any of these organisational support components (dedicated peer support training; underlying theory; support for peer support) [30, 50, 74], and four reported just one component [31, 39, 42, 68]. In contrast, four studies reported all three components [23, 35, 54, 71], and eight reported two out of three [28, 44, 45, 59, 62, 63, 66, 76].

#### Outcomes

Studies reported measuring thirteen of the eighteen outcomes of interest, with no studies of one-to-one peer support providing usable data assessing employment, symptoms of psychosis, self-stigma or experienced stigma, or emergency service use. Studies most often measured were hospitalisation [23, 28, 30, 35, 42, 45, 59, 71, 76] and quality of life [23, 28, 42, 54, 59, 62, 63, 66, 71], both measured in nine studies. We note that hospitalisation was variously measured as days in hospital, number of admissions or re-admissions, and community tenure (days spent living in the community, post-intervention, before hospital admission). Overall psychiatric symptoms were measured eight times [23, 42, 45, 54, 63, 71, 74, 76], and both of social functioning [28, 31, 35, 45, 50, 71, 74] and social network support [23, 28, 35, 42, 45, 63, 76], seven times. Given that many studies used a more general measure of functioning - i.e. the Global Assessment of Function scale [29] – we subsequently report this outcome as General and Social Functioning. Satisfaction with services [31, 35, 42, 45, 76], empowerment [50, 54, 66, 71, 74] and working alliance [23, 39, 66, 68, 74] were all measured five times. We note that some studies reported both a participant rating of working alliance with staff and a staff rating of working alliance with the participant [23, 74], while others only reported a participant rating of staff [39]. Self-rated recovery was measured in four studies [54, 63, 66, 76], with wellbeing [28, 42, 45] and engagement with services [35, 39, 66] both measured in three studies. We grouped measures of physical health (e.g. two studies separately reported scores on the physical health subscale of the Lehman Quality of Life Scale) [26] with a more general measures of wellbeing (Life Skills Profile) [36], and so we report wellbeing as Physical Health and Wellbeing going forward. Depression and anxiety were also measured in three studies, with only Seeley and colleagues [68] using a separate measure for each, Proudfoot and colleagues [50] using a generalised measure for both, and Hunkeler and colleagues [31] measuring depression only. As a result we retain Depression and Anxiety as a single outcome for the purposes of this review. Finally, hope was measured in two studies [44, 45]. Details of the specific tools used to measure these outcomes in each study can be found in Table 1 and are discussed further in the context of the meta-analysis reported below.

#### Risk of bias

**Fig. 2:**
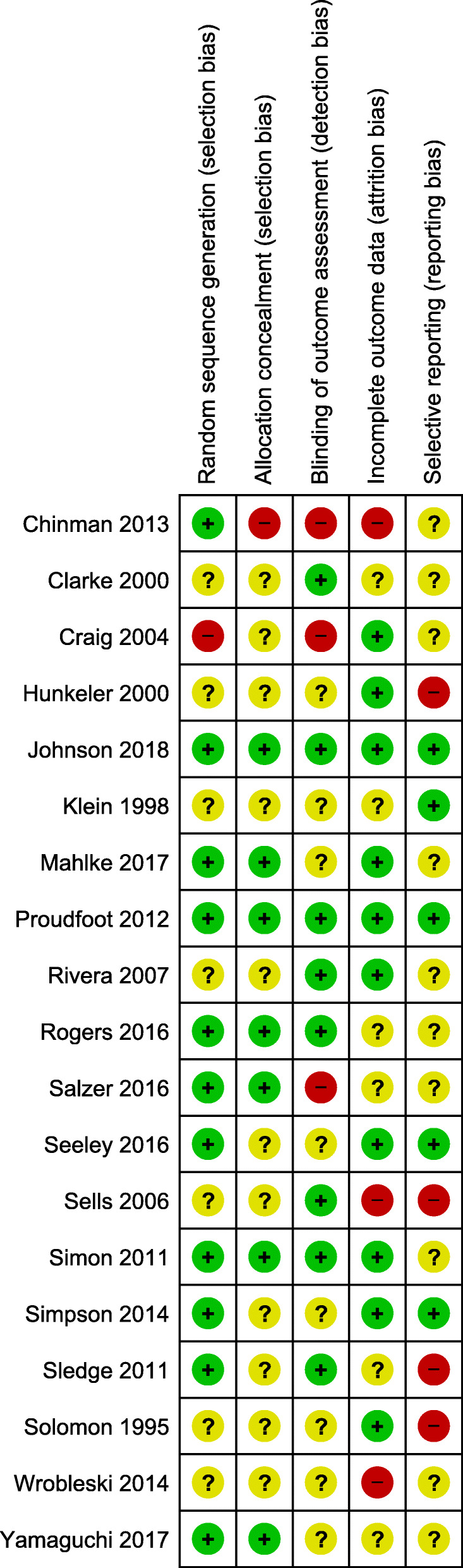
Summary of risk of bias of included studies

The Risk of Bias ratings are displayed in Fig. 2. Sequence generation was not sufficiently described in 7 of the 19 trials and was at high risk of bias in one trial. Concealment of the allocation sequence was not sufficiently described in 11 trials, and again at high risk of bias in one trial. Lack of blinding of assessors created a high risk of bias in 3 studies, and in 8 further trials it was unclear if assessors were blind. At the trial level, 3 were at high risk of bias for missing data (i.e. attrition bias) and 6 were unclear. Included studies may have measured but not reported outcomes that are included in this review; 10 with unclear description and 4 with high risk of reporting bias. Seven of the 19 studies [44, 50, 59, 63, 68, 71, 76] were at low risk of bias on at least three of the five bias categories and not high risk of bias for any category (i.e. might be described as being of low to moderate risk of bias overall), with the majority of those studies having been published since previous reviews. However on balance, overall quality of trials, when compared to previous reviews, remains low to moderate.

### Quantitative synthesis

Data were available for the meta-analysis from fourteen of the nineteen trials included in the review (sixteen papers), with two or more trials contributing to meta-analyses of nine of our original outcomes. Because of the way data were reported in the studies, we analyse these as eleven outcomes, analysing days in hospital and hospitalised as two discrete outcomes in place of hospitalisation, and separating working alliance into staff-rated and client-rated outcomes. This analysis includes data obtained from one study after contacting study authors [74]. The number of studies contributing data to each outcome included in the analyses can be seen in Table 2 below. Median length of follow-up was 12 months post randomisation, ranging from 2.5 to 24 months. In the following analyses a positive standardised mean difference (SMD) for the following outcomes - quality of life, social network support, empowerment, recovery, service satisfaction, working alliance (client and staff rated) - indicates the peer support intervention being more effective than the control condition, the opposite being the case for the following; general psychiatric symptoms, depression and anxiety, days in hospital and hospitalised.

**Table 2:** Results of the meta-analysis

**Outcome**	**FU**	**k**	**N1/N2**	**RR**	**95% CI**	**z (*p*-value)**	**I**^**2**^	**Q (*p*-value)**
Hospitalised	3–24	5	257/240	0.86	0.66, 1.13	1.1 (0.270)	38%	6.5 (0.170)
**Outcome**	**FU**	**k**	**N1/N2**	**SMD**	**95% CI**	**z (*p*-value)**	**I**^**2**^	**Q (*p*-value)**
Days in hospital	9–24	5	242/211	−0.10	−0.34, 0.14	0.8 (0.426)	39%	6.6 (0.160)
Overall psychiatric symptoms	6–24	6	440/417	−0.01	− 0.21, 0.20	0.0 (0.961)	53%	10.7 (0.057)
Quality of life	12–24	5	356/332	0.08	−0.11, 0.26	0.8 (0.424)	32%	5.9 (0.206)
Recovery	12–18	3	300/293	0.22	0.01, 0.42	2.0 (0.042	36%	3.1 (0.211)
Empowerment	6–12	4	272/247	0.23	0.04, 0.42	2.3 (0.020)	14%	3.5 (0.323)
Satisfaction with services	12–18	2	140/146	0.19	−0.05, 0.42	1.6 (0.116)	0%	0.0 (0.878)
General and social functioning	6–12	3	100/81	0.01	−0.32, 0.35	0.1 (0.937)	21%	2.5 (0.283)
Social network support	12–24	4	258/254	0.09	−0.25, 0.42	0.5 (0.602)	67%	9.2 (0.027)
Working alliance – client rated	6–24	3	112/101	0.24	−0.03, 0.51	1.7 (0.080)	0%	0.6 (0.736)
Working alliance – staff rated	6–24	2	69/70	0.15	−0.18, 0.48	0.9 (0.379)	0%	0.3 (0.594)

Key: *FU* follow-up; k – number of trials; N1 – sample size in intervention arm; N2 – sample size in control arm; *RR* Risk ratio, *SMD* Standardised mean difference, *CI* Confidence interval; z(p-value) – test of overall effect; I^2^ – measure of heterogeneity; Q(*p*-value) – Bartlett’s test of heterogeneity

#### Hospitalisation

Five trials reported the dichotomous outcome of whether hospitalised during follow-up period or not. Follow-up ranged from 3 to 24 months with data on a total of 497 participants. The risk of being hospitalised was reduced by 14% for those receiving peer support (RR = 0.86: 95% CI 0.66, 1.13). Moderate heterogeneity (I^2^ = 38%) was found across trials for this outcome. A similar result of a non-significant effect of peer support (SMD = -0.10: 95% CI -0.34, 0.14) and moderate heterogeneity (I^2^ = 39%) was found for the days in hospital outcome. The five trials in this meta-analysis had follow-up ranging from 9 to 24 months and a total sample size of 453.

#### Overall psychiatric symptoms

Six trials reported overall psychiatric symptoms with follow-up ranging from 6 to 24 months. Total sample size was 857. There was no evidence of the effect of peer support on symptoms; pooled standardised mean difference was − 0.01 (95% CI -0.21, 0.20). There was a high level of heterogeneity across trials, I^2^ = 53%, χ^2^ test of heterogeneity. Q = 10.7, *p* = 0.057.

#### Quality of life

A total of 688 participants had quality of life data reported from five trials with follow-up ranging from 12 to 24 months. No effect of peer support was found on quality of life, SMD = 0.08 (95% CI -0.11, 0.26) with moderate heterogeneity across trials, I^2^ = 32%.

#### Recovery

**Fig. 3:**
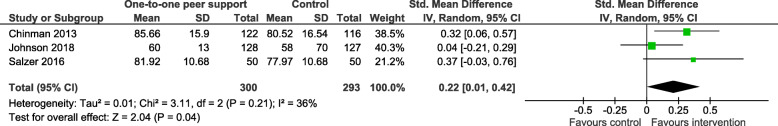
Forest plot for recovery outcome

Three trials reported appropriate recovery data with follow-up ranging from 12 to 18 months and a total sample size of 593. Peer support is shown to have a small but statistically significant benefit on recovery (SMD = 0.22: 95% CI 0.01, 0.42: *p* = 0.042) (Fig. 3). Only moderate heterogeneity is indicated, I^2^ = 38%.

#### Empowerment

**Fig. 4:**
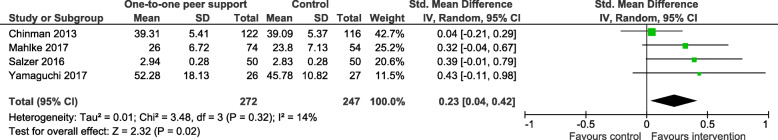
Forest plot for empowerment outcome

Four trials with a total sample size of 519 participants and follow-up ranging from 6 to 12 months reported empowerment related outcomes. Empowerment was significantly higher in those receiving peer support, a small effect size, SMD = 0.23 (95% CI 0.04, 0.42: *p* = 0.020) (Fig. 4). Heterogeneity was low, I^2^ = 14%.

#### Satisfaction with services

Satisfaction with services outcome data was available from two trials and a total of 286 participants. Follow-up in the two trials ranged from 12 to 18 months. No effect of peer support was found (SMD = 0.19: 95% CI − 0.05, 0.42) with no heterogeneity, I^2^ = 0%.

#### General and social functioning

Three trials provided data for the general and social functioning outcome on a total sample size of 181. Follow-up in the two trials ranged from 6 to 12 months. No effect of peer support was found (SMD = 0.01: 95% CI -0.32, 0.35) with little heterogeneity, I^2^ = 21%.

#### Social network support

Four trials reported social network support outcome data with follow-up ranging from 12 to 24 months and a total sample size of 512 participants. While the pooled SMD = 0.09 (95% CI -0.25, 0.42) indicated no effect of peer support on social network support, there is significant heterogeneity across the trials, I^2^ = 67%, χ^2^ test of heterogeneity. Q = 9.2, *p* = 0.027.

#### Working alliance

Client rated working alliance about staff was reported in three trials and by a total of 213 participants. Follow-up ranged from 6 to 24 months. No heterogeneity was found across trials, I^2^ = 0%, but the SMD = 0.24 (95% CI -0,03, 0.51:*p* = 0.080) indicates a potentially positive outcome for peer support. The SMD = 0.15 (95% CI -0.18, 0.48) was lower for staff ratings of the working alliance, again with no heterogeneity, I^2^ = 0%. This outcome was only rated in 2 trials, a total of 139 participants.

#### Subgroup analyses

**Fig. 5:**
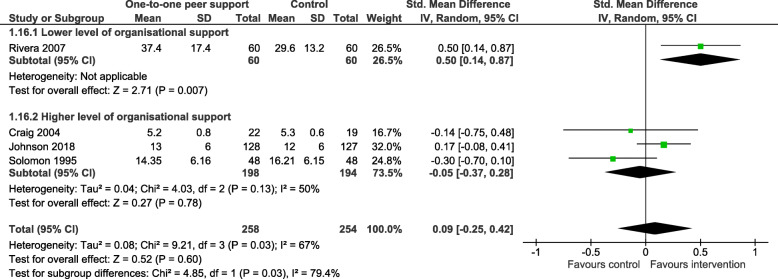
Sub group analysis; social network support by level of organisational support

**Fig. 6:**
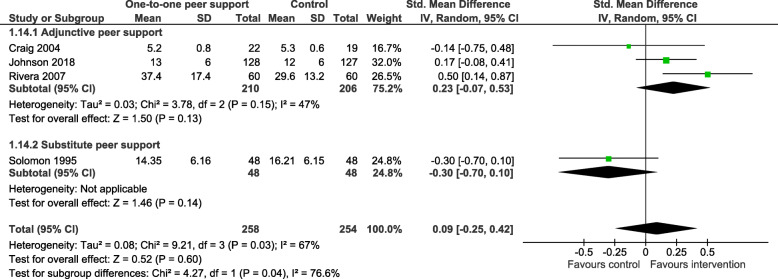
Subgroup analysis; social network support by type of peer support

Only two outcomes – overall psychiatric symptoms and social network support – satisfied our condition of sufficient heterogeneity in the data to warrant undertaking subgroups analyses (see Table 3 below). We conducted subgroups analyses of those outcomes as defined earlier: adjunctive peer support interventions compared to those where peer workers were working in a similar or substitute role to other mental health workers; studies reporting a higher level of organisational support for peer support compared to those studies reporting a lower level of organisational support for peer support. These analyses did not explain heterogeneity with respect to overall psychiatric symptoms. A single study [42], reporting a lower level of organisation support for peer support, found a moderate, significant increase in social network support for people in the peer support arm of the trial (SMD = 0.50: 95% CI 0.14, 0.87), compared to three other studies with a higher level of organisational support for peer support where no significant difference in social network support was found (SMD = -0.04: 95% CI -0.37, 0.28) (Fig. 5). It can also be seen in Table 3 that there is evidence that whether peer support is being provided as adjunctive to usual care or as a substitute role impacts the effectiveness of peer support in increasing social network support, Qint = 4.27, *p* = 0.039. The effect of peer support is significantly greater when it is delivered as an adjunctive, SMD = 0.23, as opposed to substitute intervention, SMD = -0.30, a difference of 0.53 (Fig. 6).

**Table 3:** Results of the subgroup analyses

Outcome	Subgroups	k	N1/N2	SMD	95% CI	z (***p***-value)	Qint (***p***-value)
Overall psychiatric symptoms	Substitute PS	1	48/48	0.35	-0.05, 0.75	0.1 (0.937)	3.44 (0.064)
	Adjunctive PS	5	392/369	−0.07	−0.27, 0.12	0.7 (0.466)	
	Lower level of organisational support	2	91/92	−0.24	− 0.53, 0.05	0.6 (0.521)	2.64 (0.104)
	Higher level of organisational support	4	349/325	0.09	−0.18, 0.35	0.6 (0.581)	
Social network support	Substitute PS	1	48/48	−0.30	−0.70, 0.10	1.5 (0.144)	4.27 (0.039)
	Adjunctive PS	3	210/206	0.23	−0.07, 0.53	1.5 (0.134)	
	Lower level of organisational support	1	60/60	0.50	0.14, 0.87	2.7 (0.007)	4.9 (0.028)
	Higher level of organisational support	3	198/194	−0.04	−0.37, 0.28	0.7 (0.784)	

Key: k – number of trials; N1 – sample size in intervention arm; N2 – sample size in control arm; *SMD* Standardised mean difference, *CI* Confidence interval; z(*p*-value) – test of overall subgroup effect; Qint (*p*-value) – test of subgroup differences

## Discussion

Our review has indicated that a number of additional studies of one-to-one peer support have been published in the years following previous systematic reviews, suggesting that it has become viable to consider different modalities of peer support – e.g. group, one-to-one, peer-led services – in separate reviews. Studies remain predominantly conducted in the US, but with a gradual increase in studies being conducted in Europe and beyond. With health systems operating differently in different countries, caution does need to be taken when considering any results in the round.

While this review is focused on one-to-one peer support, we still see the heterogeneity of intervention observed by Pitt [7] and Lloyd Evans [8] across modalities of peer support. However it is interesting to note that most studies of peer workers in paraclinical roles, including case-management [23, 30, 39, 42] and healthcare assistant roles [35], are now well over 10 years old, as are the majority of studies that compare peer workers to other mental health workers performing a similar role (‘substitute’ peer support) [23, 30, 39]. It is also worth noting that none of those studies of peer workers in paraclinical roles, or of peer workers in substitute roles, contributed data to analyses of those outcomes where a significant positive effect of peer support was found (recovery and empowerment).

Peer support interventions evaluated in more recent studies, in contrast, are almost exclusively evaluating adjunctive peer support, and tend to have either a structured, behavioural focus [44, 62, 68, 74, 76], or a more social focus, with peer workers providing a less structured, more peer-led support for recovery [45, 59, 63, 66, 71]. We suggest that there is potential, as more trials are published, of conducting focused reviews of specific groups or families of similar one-to-one peer support interventions.

We observe that a wide range of outcomes continue to be used. Of the original list of outcomes considered by Lloyd Evans and colleagues [8], we found that neither employment nor symptoms of psychosis were measured in the nineteen trials of one-to-one peer support that we reviewed. While Pitt and colleagues [7] found a small reduction in emergency service use for people receiving peer support we did not include data on emergency service use in our review as we excluded self-reported service use data from our analysis; Pitt and colleagues [7] themselves had suggested that recall bias and selective reporting of this outcome undermined the reliability of this particular finding.

While measures of general psychiatric symptoms were used in nearly half of all trials, measures of specific symptoms – of depression – were only used in those studies which exclusively recruited from a population diagnosed with depression [31, 50, 68]. Of our additional set of, largely, more socially-focused outcomes, neither internalised nor experienced stigma have been measured to date, although social functioning, social network support and working alliance were all measured in multiple studies, including in older trials [23, 35]. If we consider just those outcomes used in multiple studies (outcomes included in our meta-analysis), we see a more focused outcomes-set emerging, balancing clinically-orientated outcomes of general severity of symptoms, functioning and hospitalisation with a set of self-reported, psychosocial outcomes including empowerment, recovery, working alliance and social network support.

As with previous reviews, once data from multiple studies were pooled, we found no difference between peer support and control across the majority of outcomes we considered. This included hope, where Lloyd Evans and colleagues [8] found a moderate positive impact of peer support, but we note again that their review included peer support provided to groups while we found insufficient studies of one-to-one peer support reporting measurement of hope as an outcome. However, our review does suggest that trial participants offered one-to-one peer support in mental health services experience modest but significant improvement in empowerment and self-reported recovery compared to control group participants, the latter reflecting similar findings by Lloyd Evans and colleagues [8].

Studies reporting empowerment were for the most part were published since the 2013 [7] and 2014 [8] reviews, reflecting the suggestion made by Bellamy and colleagues [87] that more recent studies indicate that new peer support initiatives might usefully be directed to interventions that, broadly speaking, support individual empowerment. We grouped assessments of empowerment and related constructs together for the purposes of this review, and the studies in the analysis variously used the Patient Activation Measure [57], the General Self-Efficacy Scale [73], and the Empowerment Scale [67]. As a construct, patient activation has a clear focus on the extent to which the individual is able to access the healthcare they need, and is a good fit for interventions that specifically address the way in which the individual engages with their mental health care [54, 74]. Self-efficacy taps into the individual’s ability to make use of a wider range of support and care, while the Empowerment Scale has been shown to weight heavily on hope as a factor [67]. Again, these measures would seem appropriate for interventions focused on supporting recovery [63] and independence [66] respectively.

Studies reporting recovery as an outcome again used a range of measures. Salzer and colleagues [66] use the Recovery Assessment Scale [65], which measures recovery across five domains of personal confidence, hope, willingness to ask for assistance, goal and success orientation, and coping, and as such would seem particularly attuned to an intervention designed to support independent living. Johnson and colleagues [76] use the Questionnaire about the Process of Recovery [78], which comprises an ‘intrapersonal’ subscale that relates to “intrapersonal tasks that an individual is responsible for carrying out and that they complete in order to rebuild their life”, and an ‘interpersonal’ subscale relating to “individuals’ ability to reflect on their value in the external world and on how recovery is facilitated by external processes and interpersonal relationships with others”. Seventeen of the 22 items that comprise the measure load onto the ‘intrapersonal’ subscale, as would seem apposite for the evaluation of a self-management intervention. Chinman and colleagues [54] use the Mental Health Recovery Measure [55], measuring recovery in the seven domains of Overcoming Stuckness, Self-Empowerment, Learning and Self-Re-definition, Basic Functioning, Overall Well-Being, New Potentials, and Advocacy/Enrichment. This balance between functioning and wellbeing, and then moving on and realising potential seems well-suited to the case management function of the intervention.

These findings indicate what would seem to be an important relationship between positive impact on outcome, the assessment tool used and the intervention. As such we would suggest that trials exploring these, or indeed other outcomes, in the future should be cognisant of the constructs informing specific assessment tools (e.g. domains, subscales), and ensure that these relate closely to the mechanisms underpinning particular peer worker interventions. We reiterate calls in previous reviews [7] for a clearer understanding of the mechanisms of peer support, and the theory driven selection of outcomes that relate specifically to what peer workers do.

We note that one further outcome, client-rated working alliance, while not quite significant, demonstrated a similar effect size to the other positive outcomes. In two studies [23, 39] participants rated working alliance with peer workers in the intervention arm of the trial, compared to working alliance with mental health professional in the control arm, while in the third study [74] working alliance with a mental health professional was rated in both arms of the trial, with and without additional peer support. Once data were pooled there was a relatively small sample size for this outcome; more data would produce a more precise estimate of the effect size. This finding suggests that there is merit in exploring working alliance in future studies of one-to-one peer support, especially given other research indicating a potential mechanism for peer support in bridging and enabling connection between service users and mental health professionals [21].

We note that while both measures of hospitalisation analysed were in a positive direction (i.e. a reduction in days in hospital and risk of hospitalisation), neither were significant. The lack of positive association between the offer of peer support and reduction in psychiatric symptoms also suggests that, while studies are using a balance of clinical and more psycho-socially focused outcomes, one-to-one peer support in mental health services is unlikely to impact on clinical outcomes.

There was significant heterogeneity of data for two outcomes (overall psychiatric symptoms and social network support). While our subgroup analyses did not explain heterogeneity with respect to overall psychiatric symptoms, analyses did offer insight into the relationship between peer support and social network support. Finding that a single study [42], reporting a lower level of organisation support for peer support, indicated a moderate, significant increase in social contacts, while studies reporting a higher level of support for peer support did not, appears counter-intuitive. Looking closely, authors note that the increase in positive outcome was accounted for by additional contacts with peer workers and professional staff, rather than any increase in contacts with family or friends [42].

Furthermore, peer support that was provided in addition to care as usual was significantly more likely to increase social network support than peer support provided by peer workers employed in a substitute role. At the least, these findings suggest that it is worth considering measuring social network support in future studies, while giving consideration to how the peer support intervention might be functioning to increase social contacts. In addition, we would suggest that we have demonstrated that our approach to operationalising an analysis of organisational support for peer support is feasible and might be pursued in future reviews, subject to the availability of suitable data. Continued improvement in reporting peer support interventions might usefully include good description of the organisational support provided for peer workers [88].

While cost was not one of our outcomes of interest we note that claims have been made about the potential contribution to reducing the cost of mental healthcare that peer support might make [5]. Only one of the nineteen trials included in our review considered cost, but was not sufficiently powered to draw any conclusions [59]. As such, analysis of the cost-effectiveness of one-to-one peer support in mental health services is largely absent from the evidence base to date.

### Limitations

Overall quality of trials, when compared to previous reviews, remains low to moderate, although we note that, in our set of trials of one-to-one peer support, more recent trials appear less likely to have serious risk of bias and more likely to have low risk of bias on a majority of assessments, and so we tentatively suggest that the quality of studies is improving. Reporting bias, due to incomplete reporting of outcomes data, remains an issue and, as such, this downgrading of the quality of the overall evidence base does limit the strength of findings of this review. We note that for our two main positive outcomes, self-reported recovery and empowerment, all but one of the studies that reported measuring these outcomes included usable data in trial papers. However completeness of reporting of outcomes is essential to inform good quality evidence with respect to peer support in mental health services going forward.

In focusing on one-to-one peer support we have produced a more focused review than previous studies. However we acknowledge that studies remain heterogenous, especially with respect to clinical population (for example, only one study [71] specified chronicity of diagnosis). In addition, we note the range of terms used to describe peer support roles and acknowledge that our search might not have been wholly inclusive. Like all reviews, the validity of our study is defined by the strategy we describe above.

## Conclusions

One-to-one peer support in adult mental health services has a modest, positive effect on empowerment and self-reported recovery, and might potentially also impact on measures of working alliance between service users and mental health workers, and social network support. It seems unlikely that one-to-one peer support has a positive impact on clinical outcomes such as symptoms or hospitalisation, given data available for this review, suggesting that the benefits of peer support are largely psychosocial, operating at both individual (interpersonal) and relational (intrapersonal) levels. The quality of reporting, both of trial methods and design of peer support interventions, has improved somewhat but needs to continue to do so - especially with respect to complete reporting of outcome measurements - in order to maximise the usefulness of the evidence base for service providers and policymakers. Future trials should also consider appropriate assessment of cost-effectiveness of peer support in mental health services.

While some older trials of one-to-one peer support evaluated peer workers working in paraclinical roles, and/ or in substitute roles, newer studies focus on peer workers providing adjunctive interventions; either structured, behavioural interventions, or more socially focused, self-directed, flexible support for recovery. This review suggests that future trials of one-to-one peer support in mental health services should focus on peer workers providing interventions that are additional to usual care; outcomes for peer support are no better than control where peer workers are compared to other mental health workers doing similar work, and might be worse for outcomes such as social network support, possibly because such roles do not enable peer workers to enact a more distinctive way of working.

We suggest that studies should carefully consider the specific mechanisms of action of peer support, with trials designed so that choice of assessment tools (the constructs that are measured) reflect the specific function of the peer support intervention and the distinctive way in which peers work compared to other mental health workers. If and where peer support is having a beneficial effect, there will be a greater likelihood of observing this in a more carefully designed trial. Furthermore, as the evidence base for peer support grows it would be methodologically desirable to conduct more focused reviews of groups of similar interventions (rather than continuing to review a heterogenous group of interventions as a whole). Finally, this review demonstrated the potential to explore heterogeneity in peer support, in relation to outcome, in terms of the quality of organisational support provided to peer workers.

It is of interest to compare our findings with the concurrent review of group peer support conducted by Lyons and colleagues. We similarly identified that heterogeneity of intervention remains a feature of the evidence base while noting that a small number of types or functions of peer support are emerging (with a number of trials of peer-supported self-management identified by both reviews). Both reviews are also indicative of a modest, positive effect of peer support on self-reported recovery and an absence of effect, in the evidence to date, on clinical outcomes. Again, both reviews indicate that reporting bias – incomplete reporting of outcomes – continues to undermine the quality of the evidence base as whole.

## Supplementary Information

**Additional file 1.** Search strategy for MEDLINE, EMBASE and PsychINFO using the OVID interface.

## Data Availability

The data that support the findings of this study are available from the corresponding author upon reasonable request.

## Abbreviations

CI: Confidence Interval
DSM: Diagnostic and Statistical Manual
SMD: Standard Mean Difference
