# Supplementary material for: The effectiveness of one-to-one peer support in mental health services: a systematic review and meta-analysis
Source: BMC Psychiatry. 2020 Nov 11;20:534. doi: 10.1186/s12888-020-02923-3 (PMC7657356; doi:10.1186/s12888-020-02923-3)
Supplement: Supplementary file 1 — Additional file 1. Search strategy for MEDLINE, EMBASE and PsychINFO using the OVID interface. [file 12888_2020_2923_MOESM1_ESM.docx]

Supplementary materials 1 - search strategy for MEDLINE, EMBASE and PsychINFO using the OVID interface

| #1 | (peer* support*).mp,sh. |
| --- | --- |
| #2 | (peer* adj5 support*).mp,sh. |
| #3 | #1 NOT #2 |
| #4 | (peer* adj5 group*).mp,sh. |
| #5 | (peer* support* group*).mp,sh. |
| #6 | #5 NOT #4 |
| #7 | (community adj5 network*) OR (social adj5 network*).mp,sh. |
| #8 | #5 OR #7 |
| #9 | (peer* adj5 (provid* OR consumer* OR survivor* OR prosumer* OR specialist* OR companion*)).mp,sh. |
| #10 | (consumer* adj2 (provider* OR survivor* OR consultant*)).mp,sh. |
| #11 | ((one-to-one) adj5 support*).mp,sh. |
| #12 | (peer* adj5 (tutor* OR educat* OR mentor* OR intervention* OR listen* OR mediat* OR befriend* OR therap* OR work* OR counsel*)).mp,sh. |
| #13 | (mental health peer*).mp,sh. |
| #14 | ((peer* adj5 support*) adj5 (one-to-one OR intentional* OR formal* OR individual* OR 1-2-1)).mp,sh. |
| #15 | #14 NOT #2 |
| #16 | ((lived experience*) OR survivor* OR (service user*)).mp,sh. |
| #17 | #2 OR #9 OR #10 OR #11 OR #12 OR #13 |
| #18 | #2 OR #8 OR #9 OR #10 OR #11 OR #12 OR #13 |
| #19 | #16 AND #17 |
| #20 | #17 OR #19 |
| #21 | (mental* adj5 (ill* OR disorder* OR health OR distress*)).mp,sh. |
| #22 | ((psychiatr* discharge) OR psychiatr* OR inpatient* OR (severe* mental*) OR DSM* OR ICD-10 OR psychopath*).mp,sh. |
| #23 | (alcohol* OR dypsomania* OR (drug addict*) OR (substance abuse) OR (substance misuse)).mp,sh. |
| #24 | ((disorder* adj1 ((posttraumatic stress) OR stress OR adjustment)) OR PTSD).mp,sh. |
| #25 | ((dissociative identity disorder*) OR (dissociative amnesia) OR (depersonali#ation disorder*) OR (dereali#ation disorder*) OR (dissociative disorder*)).mp,sh. |
| #26 | (disorder* adj2 (obsessive compulsive OR body dysmorphic OR hoarding OR excoriation OR gender identity) OR OCD OR trichotillomania* OR kleptomania* OR pyromania* OR gambl* OR transsexualism OR transvestism OR (personality change*)).mp,sh. |
| #27 | ((disorder* adj2 (mood OR dysthymia OR premenstrual OR affective)) OR depress*).mp,sh. |
| #28 | ((disorder* adj2 (bipolar OR depress* OR cyclothymic OR manic)) OR (manic episode*) OR mania* OR hypomania* OR cyclothymi* OR dysthymi* OR bipolar*).mp,sh. |
| #29 | ((disorder* adj2 (schizo* OR delusion* OR psychot* OR cataton*)) OR schizo*).mp,sh. |
| #30 | (personality disorder*).mp,sh. |
| #31 | ((Disorder* adj2 (anxiety OR stress)) OR (somat* adj2 (disorder* OR dysfunction*)) OR (dissociative adj2 (disorder* OR amnesia OR fugue OR stupor OR convulsions OR anaesthesia)) OR (disorder* adj2 (panic OR obsessive compulsive OR depersonali#ation* OR neurotic)) OR hypochondri*or neurasthen* OR phobi* OR agoraphobi* OR mutism).mp,sh. |
| #32 | (anorexi* OR bulimi* OR purg* OR bing* OR EDNOS OR (eating adj2 (disorder*))).mp,sh. |
| #33 | #21 OR #22 OR #23 OR #24 OR #25 OR #26 OR #27 OR #28 OR #29 OR #30 OR #31 OR #32 |
| #34 | #20 AND #33 |
| #35 | ((mental* well-being) OR (self-esteem) OR (self-management)).mp,sh. |
| #36 | #20 AND #35 |
| #37 | #34 OR #36 |
| #38 | #37 NOT #8 |
| #39 | (Alzheimer* OR Parkinson* OR dement* OR (organic mental health)).ti,ab,sh. |
| #40 | (deliri* OR neurocognitive disorder*).ti,ab,sh. |
| #41 | #39 OR #40 |
| #42 | #38 NOT #41 |
| #43 | (cancer* OR onc*).ti,ab,sh. |
| #44 | ((heart adj2 (disease* OR failure OR attack)) OR stroke* OR hypertension). ti,ab,sh. |
| #45 | (COPD OR lower respiratory infection* OR emphysema* OR bronch* OR influenza OR pneumoni* OR asthm* OR urinary diseas*).ti,ab,sh. |
| #46 | (HIV OR AIDS OR diarrho* OR diabetes OR urinary disease).ti,ab,sh. |
| #47 | #43 OR #44 OR #45 OR #46 |
| #48 | #33 AND #47 |
| #49 | #20 AND #48 |
| #50 | #42 NOT #47 |
| #51 | #49 OR #50 |
| #52 | (infan*or child* OR youth* OR adolescen* OR school* OR girl* OR boy*).ti,ab,sh. |
| #53 | (animal* OR primate* OR monkey* OR gibbon* OR horse* OR rat* OR mouse OR mice).ti,ab,sh. |
| #54 | #52 OR #53 |
| #55 | #51 NOT #54 |
| #56 | #2 AND #55 |
| #57 | (peer* adj5 (provider* OR counsel* OR specialist* OR run OR intervention* OR work* OR led OR wellness OR educator* OR assistance OR co-lead* OR leader* OR mentor OR consumer* OR tutor* OR mediat*)).ti,ab. |
| #58 | #55 and #57 |
| #59 | #56 OR #58 |
